# Language Learning Motivations among Turkish Learners of Chinese as a Foreign Language: A Survey of Five Universities in Turkey

**DOI:** 10.3390/bs13100808

**Published:** 2023-09-29

**Authors:** Yake Wang, He Yang

**Affiliations:** 1 Institute of Education, Xiamen University, Xiamen 361005, China; wangyake@stu.xmu.edu.cn; 2 College of Foreign Languages and Cultures, Xiamen University, Xiamen 361005, China; 3 Institute of Foreign Languages and Literatures, Xiamen University, Xiamen 361005, China

**Keywords:** learning motivation, Turkish learners, Chinese as a foreign language, integrativeness, instrumentality

## Abstract

Research on language learning motivations has been extensive. However, research on learners’ motivations for learning Chinese has been underexplored compared to that for learning English. The current study aimed to investigate the motivations among 256 Turkish learners of Chinese as foreign language (CFL) who studied at five universities in Turkey. Participants completed an adapted questionnaire based on Gardner’s Attitude/Motivation Test Battery (AMTB). A series of statistical analysis revealed three major findings. First, integrativeness, attitudes towards learning situation and attitudes towards learning Chinese were identified as the three most important motivational variables, followed by instrumentality, and parental encouragement. Language anxiety and passive motivation seemed to play a weaker role in Turkish CFL learners’ motivations for learning Chinese at the tertiary level. Second, the results also show that females exhibited higher motivation to learn the target language compared to their male peers. Third, the choice of major among CFL learners appeared to influence their language learning motivations, with a notable distinction between Chinese majors and non-Chinese majors in five motivational variables: integrativeness attitudes towards the learning situation, language anxiety, parental encouragement, and passive motivation.

## 1. Introduction

Motivation is the drive behind the reasons we act in certain ways, determining the behaviors of humans. Over the past decades, research on language learning motivation has attracted much attention in the field of second language (L2) acquisition. However, more than 70% of the studies on L2 learning motivation have predominantly focused on English as the target language [1]. At present, as a result of economic globalization, many countries and regions are actively promoting multilingualism among young people to foster openness and multiculturalism. There is no doubt that the “Multilingual Turn” has also significantly influenced research on motivation for L2 learning. Consequently, the question arises as to whether the theories regarding motivation for learning an L2 are applicable to languages other than English. To answer this question, it is essential for researchers to focus on language learning motivation of learners from different cultural backgrounds and their L2 learning experiences.

China’s rapid economic growth and expanding global influence have propelled the Chinese language to the forefront of international discourse. As a result, the Chinese language has emerged as one of the world’s most important languages [2]. While previous studies on foreign language learning motivation have primarily focused on major languages such as English, relatively scant attention has been given to the motivation for learning Mandarin Chinese [3]. Due to the growing economic, cultural, and educational cooperation between China and Turkey, Turkish university students are becoming increasingly interested in the Chinese language and culture, leading to a steady growth in the number of students studying Chinese [4]. However, there is dearth of research on Turkish university students’ motivations for learning Chinese. An interesting yet understudied opportunity exists to investigate motivation in the context of Chinese learning in Turkey. Therefore, this paper aimed to examine L2 motivation of Turkish university students while learning Chinese as an L2. It intended to provide a structured depiction of the motivational traits exhibited by Turkish learners of Chinese as a foreign language (CFL). It also investigated the effect of gender and major on learners’ L2 motivation and examined how these factors affected their learning outcomes. We conducted a survey in five universities in Turkey, and a total of 256 Turkish students responded to the questionnaire. Their responses allowed us to identify the general trends and interesting contrasts in their motivation to learn Mandarin Chinese. 

Turkey is a country strategically located at the crossroads of Eurasia, with an important geographical position and a wealth of cultural heritage. This study will offer valuable insights into the motivations of Turkish CFL learners. Moreover, it can contribute to a better understanding of the factors that influence language learning in a cross-cultural context. It will expand the specific scope of Chinese language learning motivation in different countries and enrich the overall study ecology. Finally, this research can help inform curricula and pedagogical practices in Turkish universities.

## 2. Literature Review

### 2.1. Gardner’s Socio-Educational Model

Gardner and MacIntyre [5] developed the Socio-Educational Model of second language acquisition (SLA), which suggests that antecedent factors, individual differences, and second language acquisition context influence L2 learning outcomes. According to Dörnyei [6], individual differences account for a great deal of variance in L2 attainment and are the most consistent predictors of L2 learning success. Individual differences include intelligence, language aptitude, learning strategies, attitudes, motivation, and anxiety [5]. According to Gardner [7] (p. 6), students’ attitudes toward a language group influence their success in learning the language. In Gardner’s theory of L2 motivation, integrative motives are considered, along with a general learning model derived from them, known as the socio-educational model, and the Attitude/Motivation Test Battery (AMTB), a widely used standardized instrument with well-designed psychometric features. The AMTB has been extensively employed in empirical studies to assess individual differences in motivations among learners of various languages. It is also used to assess the effectiveness of language teaching methods. The AMTB has been found to be a reliable and valid tool for measuring L2 motivation. With its concept of integrativeness, Gardner’s motivation theory has significantly influenced L2 motivation research.

### 2.2. Motivation for Learning Chinese as a Foreign Language

The field of L2 motivation research has thrived for several decades, but a significant majority of previous studies, over 70% of them, have predominantly focused on the acquisition of global English [1]. Thus, there is a relatively small body of research on learners’ motivation to learn languages other than English. With the growing promotion multilingualism and the preservation of linguistic and cultural diversity [8,9], a new area of research has emerged to examine language learners’ motivation to learn languages other than English. For example, there is a small but increasingly growing body of studies examining CFL learners’ motivation for learning Mandarin Chinese [10,11,12,13].

As China’s economy continues to develop, the number of individuals learning the Chinese language has significantly increased, while the ethnic diversity of learners has also widened [14]. Research on foreign students studying in China has revealed that a strong integrative motivation is critical to positive socio-cultural adaptation and academic adjustment [15]. Additionally, the strength of this motivation tends to grow with the duration of studying in the target language country [11,16], as observed in the case of Arab learners in China who demonstrated a high level of integrative motivation to learn Chinese [10] and in the case of New Zealand students who demonstrated the dynamic nature of CFL motivation during their study in China [11]. Initially, Asian students in the United States are motivated to learn Chinese due to their inherent interest in Chinese culture and their desire to learn about their cultural heritage [17].

Lee [18], on the other hand, discovered that Chinese language learning is usually prioritized for functional and practical reasons, known as instrumental motivation. For instance, foreign language learners in the Philippines tend to be more inclined towards instrumental motivation than integrative motivation [12]. In Hong Kong, South Asian students’ motivation to learn Chinese is entirely driven by practical purposes [19]. A comparable situation exists in India, where instrumental motivation is the sole determinant in learning Chinese as a third language [13]. Additionally, for Australian learners, instrumental motivation is their primary inclination in studying Chinese [8].

Danish studies suggest that learners’ intrinsic motivation is increased by their affective and learning contextual factors [20]. Similarly, statistical analysis in the U.S. context confirms the interplay between motivation and the learning environment during the learning process [21]. Additionally, CFL motivation was found to affect Thai learners’ Chinese language proficiency while taking online Chinese courses [22].

### 2.3. Gender and L2 Motivation

Earlier studies suggested that language learners’ gender played a significant role in their motivation to learn a second language [8,23,24,25,26,27,28]. For example, Kissau et al. [25] reported that there was less motivation among male high school students to learn Spanish than among female students, although there were no significant differences. Similarly, a mixed-methods study investigating the effect of gender on motivation to learn English revealed significant gender differences in Turkish university students’ motivation levels [26], with female students demonstrating higher levels of motivation than their male peers. The study conducted by You and Dörnyei [28] examined motivational profiles of English language learners in Chinese secondary schools and universities. According to their study, female learners performed better on all motivational scales except for the ought-to L2 self dimension and intended learning efforts. By the same token, Guo and Zhou [24] observed a similar pattern among English language learners in China, where females exhibited higher levels of intrinsic motivation, extrinsic motivation, and test anxiety, and placed a stronger emphasis on communication, connectedness, and self-efficacy compared to males.

In certain Asian countries, such as Kazakhstan and Uzbekistan, women showed greater motivation for learning foreign languages than men, whether the perceived benefits were social, work-related, or cognitive and neurological [23]. More recently, Yang [27] found that the gender of Chinese university students significantly influenced their motivation to learn English. In all motivation measures, female students performed significantly better than male students. Males and females differed primarily in their attitudes towards the L2 community and toward learning English. Regarding Chinese language learners in Australia, females exhibited a stronger instrumental orientation compared to males [8]. While previous L2 motivation research has focused on English language learning, little is known about the effect of gender differences on motivation for learning Mandarin Chinese.

### 2.4. Major and L2 Motivation

The choice of major can potentially affect learners’ L2 motivation. It is generally believed that university students majoring in foreign languages have different motivational profiles compared to those who learn languages as minors, primarily due to the differences in the amount of target language-related instruction they receive. Additionally, specialized and mandatory courses are more likely to motivate students, as they are perceived as essential for future careers. For example, individuals majoring in English often pursue careers where the use of English is crucial. Consequently, these students have a personal stake in acquiring the language, which enhances their level of proficiency and fosters a more positive outlook on their learning outcomes compared to their counterparts who do not major in English. Over the past decade, several studies have examined the role of major in language learning motivation, yielding diverse results [27,28,29,30].

You and Dörnyei [28] revealed that parental expectations were significantly higher for university English majors compared to non-English majors. This indicates that parental expectations significantly increase when it comes to students’ principal degree course and their future careers. In a quantitative study conducted in Vietnamese universities, Ngo et al. [29] investigated the motivation and effort expended by English majors and non-English-major students in learning English. The results showed that both groups were highly motivated to learn English as part of their career preparation. Furthermore, the English majors felt less obligated to learn English and more intrinsically motivated.

Yang [27] investigated Chinese university students’ motivations to learn English. A total of 508 freshmen responded to the motivation questionnaire. Surprisingly, the results revealed that there was no significant difference in overall L2 motivation scores among English majors, arts majors (excluding English majors), and science majors, suggesting that students from all majors had similar motivations for studying English. Further analysis found that students’ ideal L2 self was the only motivational variable significantly influenced by major, while the other six motivational variables showed no significant influence. 

A qualitative study conducted by Sun, Teo, and Wang [30] examined the effect of majors on Chinese learners’ motivation for learning English. The study involved ten mature learners (older than university students), and data were collected through semi-structured interviews. The results showed that English majors tended to be goal-oriented, while non-English majors tended to be means-oriented. English majors also displayed additional integrative motivations. Furthermore, English majors had a broader range of factors influencing their motivation compared to their peers who were not English majors.

Several common findings can be observed in the review above. First, the existing L2 motivation research has primarily focused on English learning, leaving L2 motivation for languages other than English underexplored. Second, students’ motivation to learn a foreign language may vary by gender, with female students exhibiting higher levels of motivation compared to their male counterparts. Third, L2 motivation among university students is affected to varying degrees by major. However, few studies have examined university students’ motivation for learning Mandarin Chinese, especially in the Turkish context. Furthermore, there is a need for in-depth investigations into how gender and major influence learners’ L2 motivation.

## 3. Rationale for the Current Study

It is clear from the literature that language learning motivation among CFL learners from diverse L1 backgrounds has not been studied extensively. Moreover, there have been relatively few studies on Chinese language learners outside mainland China [31], particularly in less developed countries [22]. The available literature shows that there is currently limited understanding of Turkish university students’ L2 motivations for learning Mandarin Chinese. Empirical studies exploring Turkish learners’ motivations for learning Chinese are scarce, underscoring the significance of this area for future research. Additionally, while existing studies have investigated the motivational orientations among CFL learners, there is a dearth of research exploring the impact of CFL learners’ individual variations in motivations to learn Mandarin Chinese. Therefore, the present study aimed to examine L2 motivation among Turkish CFL learners, with a specific focus on exploring the potential roles that gender differences and majors may play in shaping their L2 motivation. Based on the abovementioned theoretical and empirical descriptions, the following three research questions (RQs) were posited:

- RQ1: What is the current state of language learning motivation among Turkish CFL learners?
- RQ2: To what extent does gender influence Turkish CFL learners’ motivations for learning Mandarin Chinese?
- RQ3: What role does the choice of major (i.e., Chinese as a major course or non-major course) play in shaping Turkish CFL learners’ motivation for learning Mandarin Chinese?

## 4. Methods

The research employed a cross-sectional design and utilized a self-report survey administered to adult students across five universities.

### 4.1. Participants

The sample for this study consisted of university students from Turkey, with a total of 256 participants. Among them, 108 were male (42.2%) and 148 were female (57.8%), ranging in age from 18 to 37 years. The participants were recruited from five different universities in Turkey to ensure greater representativeness of the sample [32]. These universities included a private foundation university and four public universities. Geographically, three universities were located in Ankara, one in Istanbul, and one in Kayseri. Chinese was offered as a degree program at the university in Kayseri, whereas it was offered as an elective course at the other four institutions. Among the participants, 101 (39.5%) were Chinese majors, while 155 (60.5%) were not. Table 1 presents the universities, genders, and majors of the samples. All the participants had studied Chinese for at least one semester.

### 4.2. Instrument

Motivation is a latent construct that cannot be directly observed or measured [33]. Therefore, in line with the majority of previous studies, a self-report questionnaire was utilized in the present study. The Chinese Learning Motivation Questionnaire used in this study was modified and adapted from Attitude/Motivation Test Battery [34]. The reason for adopting an adapted version instead of the standard one was due to the different participants and different target languages involved. The questionnaire consisted of 54 items that reflected the core concepts of Gardner’s [7] motivational theory. These items were grouped into seven categories: attitudes towards learning Chinese, integrativeness, instrumentality, attitudes towards the learning situation, language anxiety, parent encouragement, and passive motivation. All items were rated on a 7-point Likert scale (1 = strongly disagree, and 7 = strongly agree). Table 2 presents the mean value, standard deviation and the Cronbach alpha of each motivational variable, while an overview of the motivation questionnaire items and their factor loadings can be found in Table 3.

In addition to the main part, the questionnaire also included items focusing on participants’ demographic information, such as age, gender, nationality, and learning duration.

### 4.3. Data Collection Procedure

The research was conducted in five Turkish universities that offered Chinese language courses to both undergraduate and postgraduate students. Among these universities, one provided a degree program in Chinese, while the other four offered Chinese as elective courses. Initially, a pilot study was executed in one of the universities, involving approximately 40 participants who had volunteered for the study. After collecting responses from the participants and conducting a preliminary data analysis, certain questionnaire items were excluded or modified. Ultimately, a total of 54 items were retained. An exploratory factor analysis was conducted by the researchers to determine the validity of the questionnaire items.

Next, the first author of this paper distributed printed copies of the questionnaire to each university, either by mail or through hand delivery. Additionally, an administration manual was provided to the teachers involved in disseminating the questionnaire. The manual outlined the following guidelines on the information to be provided to students before the survey, the procedure for administering the questionnaire, and the measures to be taken in case of any problems that might occur during the distribution and completion of the questionnaire by students. In each university, there was a designated contact person responsible for distributing and collecting the questionnaires and completing the relevant forms. Moreover, the Project Information Sheet and Consent Form were distributed to all potential participants to seek their permission to use the elicited data. Participants willingly volunteered to take part in the study.

Then, 10 Chinese language teachers from the five universities were invited to assist with the study. They distributed the Chinese Learning Motivation Questionnaire to all participants present in the classroom. Participants were asked to answer all questionnaire items during the class. On average, it took 15 minutes to complete all items. Once completed, all questionnaires were collected by the research team. The raw data were entered into a computer using Microsoft Excel by the researcher. Finally, the data files were converted to SPSS format and saved by the researcher.

### 4.4. Data Analysis

The SPSS version 24.0 was used for the statistical analysis of the data. The motivation questionnaire answers were coded on a scale of 1 (strongly disagree) to 7 (strongly agree). Reliability analysis confirmed the reliability of the scales. Moreover, we coded males as “1” and females as “2”, and Chinese majors as “1” and non-Chinese majors as “2”, as Turkish CFL learners’ genders and majors were considered independent variables in the current study. The principal statistical procedures used for analysis of the data were exploratory factor analysis and independent sample *t*-test.

## 5. Results

### 5.1. Turkish CFL Learners’ Motivational Orientations for Learning Mandarin Chinese

Table 2 presents the descriptive statistics of the seven motivational variables as measured in the entire sample and in all the sub-samples. The purpose of providing such a comprehensive summary of the results was to enable the dataset to serve as a baseline for comparisons in future investigations. In terms of the entire sample, the mean values ranged from 3.34 to 6.11 on a 7-point scale, and on six of the seven scales, they exceeded the midpoint of 3.50, indicating a generally favorable disposition towards learning Chinese language. This was also evidenced by the highest scale value obtained for integrativeness (*M* = 6.11), attitudes towards the learning situation (*M* = 5.91), attitudes towards learning Chinese (*M* = 5.64). Only one scale passive motivation was below the midpoint (*M* = 3.34). The mean value of all subscales was 5. It could be tentatively concluded that our participants in general held a positive attitude towards learning Chinese as a foreign language.

Before proceeding to further statistical analysis, the motivation questionnaire was evaluated for reliability and validity. Internal reliability was measured using Cronbach’s Alpha Coefficient [35]. Based on the results, the dataset could be used for further statistical analyses (α = 0.73). Moreover, Table 2 presents the results of the reliability tests for each subscale. Furthermore, an exploratory factor analysis was conducted to enhance the validity of our findings. Firstly, the results of the Kaiser–Meyer–Olkin (KMO) test and Bartlett’s test showed a strong correlation among variables (KMO = 0.80; *p* = 0.000 < 0.01), indicating that factor analysis could be conducted for these datasets. Next, to identify the number of significant factors, an initial eigenvalue analysis was conducted. The results revealed 15 factors consisting of a total of 35 items with an eigenvalue greater than 1 (see Table 3), and they accounted for 67.50 of the total. The items with values smaller than 1 were eliminated (*n* = 19) based on Kaiser’s Criterion [36], as they did not have significant correlations with any of the factors. The scree test of Eigenvalues confirmed the same number of factors (*n* = 7) to have values greater than 1. The loadings of the factors are presented in Table 3.

### 5.2. Comparison of L2 Motivation across Genders

Table 4 presents the results of independent sample *t*-tests, revealing significant differences between male and female learners in overall motivation for learning Chinese (*t* (254) = −4.45, *p* < 0.05). In particular, the motivation of male learners (*M* = 4.80, *SD* = 0.61) was significantly lower than that of female learners (*M* = 5.14, *SD* = 0.59).

**Figure 1:**
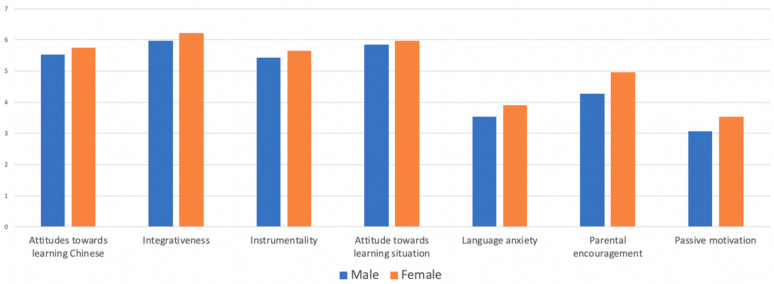
Mean values of motivational subscales across genders.

As displayed in Figure 1, the female CFL learners’ mean values of the seven subscales were higher than their male peers. For both male and female students, the top three motivational variables were integrativeness (*M_m_* = 5.96, *M_f_* = 6.22), attitudes towards the learning situation (*M_m_* = 5.85, *M_f_* = 5.97), and attitudes towards learning Chinese (*M_m_* = 5.52, *M_f_* = 5.74). Moreover, passive motivation (*M_m_* = 3.06, *M_f_* = 3.54) and language anxiety (*M_m_* = 3.53, *M_f_* = 3.91) were the lowest two motivational variables among all Turkish CFL learners.

As shown in Table 5, the results of the independent sample *t*-test showed that a significant difference was observed between male and female learners on parental encouragement (*t* (254) = −3.84, *p* < 0.01). This suggests that female learners were more likely to receive encouragement from their parents (*M* = 4.97, *SD* = 1.44) compared to their male peers (*M* = 4.28, *SD* = 1.42). Additionally, there was a significant difference in integrativeness between male and female learners (*t* (254) = −2.796, *p* < 0.01), indicating that female learners exhibited a stronger inclination towards integrating with L2 community than their male peers. Moreover, it appeared that integrativeness was the most powerful motivation for both male learners (*M* = 5.96, *SD* = 0.83) and female learners (*M* = 6.22, *SD* = 0.61) to learn Mandarin Chinese. 

Further, a significant difference was also found in language anxiety (*t* (254) = −2.435, *p* < 0.05), suggesting that female learners (*M* = 3.91, *SD* = 1.33) exhibited a higher level of language anxiety compared to their male counterparts. Finally, there was a statistically significant difference between genders in terms of their attitudes towards learning Chinese (*t* (254) = −2.403, *p* < 0.05) and passive motivation (*t* (254) = −2.136, *p* < 0.05). These findings suggested that our female participants held a more positive attitude towards learning Chinese and stronger passive motivation than their male counterparts. 

The results of the statistical analysis revealed that there was no significant difference in the mean values for instrumentality (*t* (254) = −1.77, *p* = 0.078 > 0.05). The females’ scores (*M* = 5.64, *SD* = 0.92) were slightly higher than the males’ (*M* = 5.43, SD = 0.93), but this difference was not significant. The results also revealed that both males (*M* = 5.85, *SD* = 0.98) and females (*M* = 5.97, *SD* = 0.86) demonstrated a more positive attitude towards the learning situation. Although female students had a slightly higher scores, there was not a statistically significant difference between genders in terms of their attitudes towards the learning situation.

To summarize, although the mean values of female learners were higher than those of male learners across all seven motivational subscales, the significant differences were found between male and female learners in five subscales: parental encouragement, integrativeness, language anxiety, attitudes towards learning Chinese, and passive motivation. On the other hand, there were no significant differences between genders in the other two subscales examining instrumentality and attitudes towards the learning situation.

### 5.3. Comparison of L2 Motivation across Majors

Table 6 presents the results of independent sample *t*-tests, revealing significant differences between Chinese majors and non-Chinese majors in overall motivation for studying Chinese (*t* (254) = − 4.62, *p* < 0.01). Specifically, Chinese majors were significantly more motivated compared to non-Chinese major students. In comparison with non-Chinese majors (*M* = 4.86, *SD* = 0.58), Chinese majors’ average L2 motivation score (*M* = 5.20, *SD* = 0.62) was significantly higher.

**Figure 2:**
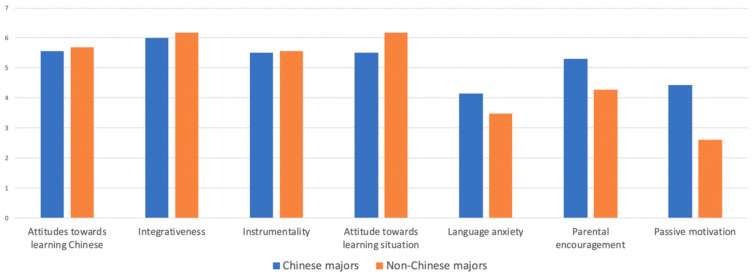
Mean values of motivational subscales across majors.

As displayed in Figure 2, for Chinese majors, the top three motivational variables are integrativeness (*M* = 6.00), attitudes towards learning Chinese (*M* = 5.57), attitudes towards the learning situation (*M* = 5.51). For non-Chinese majors, integrativeness (*M* = 6.19) is at the top, followed by the attitude towards the learning situation (*M* = 6.18) and the attitudes towards learning Chinese (*M* = 5.70). Notably, these variables are different from those of Chinese majors. To summarize, integrativeness is the strongest motivation for both Chinese majors and non-Chinese majors. Moreover, Chinese majors (*M* = 5.31/4.44/4.15) outscored non-majors (*M* = 4.27/2.62/3.49) on three subscales: parental encouragement, passive motivation, and language anxiety. Additionally, Chinese majors’ mean scores were lower than non-Chinese majors on the other four motivational subscales.

The results of the independent sample *t*-tests also revealed significant differences between Chinese majors and non-Chinese majors in the subscales of integrativeness (*t* (254) = 2.08, *p* < 0.05), attitudes towards the learning situation (*t* (254) = 5.79, *p* < 0.01), language anxiety (*t* (254) = −4.29, *p* < 0.01), parental encouragement (*t* (254) = −6.26, *p* < 0.01), and passive motivation (*t* (254)= −9.29, *p* < 0.01). On the other hand, there were no significant differences between Chinese majors and non-Chinese majors in the subscales examining attitudes towards learning Chinese and instrumentality. It has been determined that there exists a significant disparity in parental expectations and passive motivation between Chinese majors and non-Chinese major students. Some students chose to major in Chinese to meet their parents’ expectations, indicating that parental expectations play a significant role in shaping students’ future career choices. Consequently, the Chinese-major group exhibited a greater degree of language anxiety than the non-major group (Table 7).

## 6. Discussion

### 6.1. Turkish CFL Learners’ Motivation for Learning Mandarin Chinese

The first research question in the study focused on students’ motivational orientation for learning Mandarin Chinese as a foreign language in the Turkish context. The quantitative data revealed that seven motivational variables influenced learners’ motivation to learn Mandarin Chinese at Turkish universities. The three most important motivational variables were integrativeness (i.e., making Chinese friends and interacting with the target language communities), attitudes towards the learning situation, and attitudes towards learning Chinese, followed by instrumentality and parental encouragement. Turkish university CFL learners exhibited highly integrative motivation (*M* = 6.11), indicating a keen interest in the L2 culture and a positive attitude towards L2 community. Similarly, Xu, Zhang, Sukjairungwattana, and Wang [22] reported Thai CFL learners exhibited a strong intrinsic motivation (i.e., attitudes towards learning Chinese) to learn Chinese online during the COVID-19 pandemic. However, the findings of this study were not consistent with those of the previous studies [12], which showed that instrumentality, especially job prospects, was the primary motivation for university students to learn an L2. The divergence in findings can be attributed to two main reasons. Firstly, from the students’ perspective, regardless of whether they were taking the Mandarin Chinese language course as a major or not, participants in the current study generally showed a great interest in China, its culture, or its people. Second, in terms of language learning environments, there were more than 12 native-Chinese-speaking teachers providing instruction at the five universities, and a wide range of events related to the Chinese language or Chinese cultural (e.g., China Day, Chinese festival celebrations, Chinese-language competitions, summer camps, and Chinese film exhibitions) had been organized. This authentic Chinese learning environment provided Turkish CFL learners with ample opportunities to communicate in the target language, which in turn enhanced their enthusiasm for learning Mandarin Chinese.

### 6.2. The Effect of Gender Differences on Turkish CFL Learners’ L2 Motivation

The second research question concerned the effect of gender differences on learners’ motivation to learn Chinese. The results show that overall L2 motivation was also significantly higher among female CFL learners compared to their male counterparts in the Turkish context, suggesting that gender played a significant role in L2 motivation. Studies like Kissau et al. [25] and Yang [27] also found similar results. Kissau et al. [25] revealed that female high school students displayed greater motivation to learn Spanish than their male peers. Yang [27] also found that female university students exhibited a higher level of motivation than their male counterparts while learning English as a foreign language.

An investigation of gender differences on seven motivational variables revealed significant differences in five of them (parental encouragement, integrativeness, language anxiety, attitudes towards learning Chinese, and passive motivation) among Turkish CFL learners. These findings are consistent with those of previous research on motivation, which showed that male students exhibited less intrinsic and extrinsic motivation and test anxiety compared to their female counterparts [8,24]. Additionally, the most significant difference between male and female CFL learners was parental encouragement, with female learners scoring significantly higher than their male peers in the motivational variable. These finding suggest that parental encourage is an important source of motivation for female Turkish CFL learners to learn Mandarin Chinese. The findings of this study are consistent with those of previous empirical studies on L2 motivation and gender, including Mori and Gobel [37] and Okuniewski [38], which found that female learners exhibited a more positive attitude toward their L2 community compared to their male learners. This difference in motivation may be attributed to gender-based socialization. Therefore, the results suggest that educators should take gender differences into consideration when designing their teaching approaches and employ more tailored strategies for each gender.

### 6.3. The Effect of Majors on Turkish CFL Learners’ L2 Motivation

The third research question focused on the influence of major on CFL learners’ motivation to learn Chinese. The results revealed that Chinese majors were significantly different from non-Chinese majors in overall motivation for studying Chinese. Specifically, students majoring in Chinese were significantly more motivated than non-Chinese major students, suggesting the choice of major plays a significant role in L2 motivation. The results from the current study are in line with findings from the previous studies, such as Ngo et al. [29] and Sun, Teo and Wang [30]. According to Ngo et al. [29], Vietnamese students majoring in English were more intrinsically motivated and invested more effort in learning English than non-English majors. However, the results of this study differ from those of a previous study [27], which found no significant difference in overall L2 motivation scores among English majors, arts majors (excluding English majors), and science majors.

An investigation of differences in majors on seven motivational variables revealed significant differences in five of them (integrativeness, attitudes towards the learning situation, language anxiety, parent encouragement, and passive motivation) among Turkish CFL learners. These findings are partly in line with the previous findings of motivation research, which showed that parental expectations were significantly higher for university English majors compared to non-English majors [28]. This indicates that in both Chinese and Turkish contexts, parental expectations significantly increase when it comes to students’ principal degree course and their future careers.

Furthermore, this study did not find any significant difference in motivational subscales, such as attitudes towards learning Chinese and instrumentality. These results align with the previous research conducted by Yang [27], where six motivational variables, including cultural interest, attitudes toward the L2 community, attitudes towards learning English, intended learning effort, ought-to L2 self, and instrumentality, were not significantly influenced by major. It was demonstrated that both groups shared comparable attitudes towards learning Chinese and were immensely driven to acquire Chinese language skills as a constituent of their occupational groundwork.

### 6.4. Implications and Limitations

The findings have implications for teaching Chinese in a foreign language context. Firstly, teachers should strive to clarify learners’ motivational orientations and create more opportunities to enhance motivation. For instance, teachers could design more activities that allow students to interact with the Chinese community and its culture, thereby enhancing their integrativeness and intrinsic motivation. Moreover, teachers should provide appropriate instruction in learning strategies to better support students based on the effects of gender on motivation. In addition, classroom learning needs to be supplemented with informal education outside of the classroom. To promote sustainable Chinese language learning among CFL learners, educational stakeholders need to facilitate authentic communication and provide necessary resources [39]. Further research is warranted to gain a better understanding of the underlying mechanisms of CFL learners’ language acquisition. Moreover, it is important to explore the potential of digital learning environments and new technologies in supporting Chinese-language teaching and learning.

However, there are a number of issues that need to be addressed in future research. Although collecting data from five different universities allowed us to maximize the diversity of the sample, our conclusions may not be applicable to all Turkish CFL learners, especially those at an advanced level of Chinese proficiency. Moreover, although we examined the role of gender and major on CFL motivation, our sample size restricted us to the exploration of the relationship with one motivational subscale at a time. Furthermore, it would not be sufficient to analyze means descriptively to demonstrate the motivation orientations of Turkish CFL learners. The survey only reveals which motivational subscale is more or less prevalent among respondents, and qualitative data can be incorporated to capture the dynamics of motivational development [11] and the factors that drive it. Nevertheless, our findings can serve as a starting point for CFL motivation research in the Turkish context, which might help to inform and improve curriculum design and pedagogical practices in the universities of developing countries.

## 7. Conclusions

The present study has investigated Turkish university students’ motivation for learning Mandarin Chinese. It also examined the effect of learners’ gender differences and majors on their L2 motivation. The findings indicate that the motivation of Turkish CFL learners to learn Chinese is driven by seven components. Among these components, integrativeness, attitudes towards the learning situation, and attitudes towards learning Chinese were found to be the top three motivational variables for Turkish university students to learn the target language. Moreover, gender differences had an important role in Turkish CFL learners’ L2 motivation, with female learners being more motivated to learn Mandarin Chinese compared to their male peers. In addition, CFL learners’ majors might affect their language learning motivation, with a notable distinction between Chinese majors and non-Chinese-major students in five motivational variables: integrativeness, attitudes towards the learning situation, language anxiety, parental encouragement, and passive motivation. The findings suggest that studies conducted in different language learning contexts focusing on languages other than English can contribute to a deeper understanding of language learners’ motivations for learning the target language.

## Figures and Tables

**Table 1:** Number of valid samples.

		*n*	%
University	A University, Ankara	66	25.8
	B University, Ankara	38	14.8
	C University, Ankara	26	10.2
	D University, Istanbul	25	9.8
	E University, Kayseri	101	39.5
Gender	Male	108	42.2
	Female	148	57.8
Major	Chinese majors	101	39.5
	Non-Chinese major students	155	60.5

**Table 2:** Reliability estimates for L2 motivation questionnaire (*n* = 256).

Subscales	Number of Items	Mean	Standard Deviation	Cronbach’s Alpha
Attitudes towards learning Chinese	12	5.65	0.76	0.73
Integrativeness	12	6.11	0.72	0.80
Instrumentality	7	5.55	0.93	0.65
Attitudes towards the learning situation	8	5.92	0.91	0.77
Language anxiety	8	3.75	1.25	0.76
Parental encouragement	4	4.68	1.47	0.76
Passive motivation	3	3.34	1.77	0.70

**Table 3:** Items and factor loadings for each motivational variable in the questionnaire (*n* = 256).

Subscales (Factor)	Questionnaire Items	Loading
1. Attitudes towards learning Chinese	3. When I am studying Chinese, I ignore distractions and focus on my task.	0.56
	6. Learning Chinese gives me a sense of accomplishment.	0.60
	17. I work on my Chinese skills almost every day to stay up to date.	0.71
	20. I genuinely enjoy learning Chinese.	0.64
	37. I am learning Chinese because I want to spend some time in China.	0.77
	49. I put in a lot of effort to learn Chinese.	0.58
	52. I have a desire to learn as much Chinese as possible.	0.50
2. Integrativeness	1.I wish I could speak multiple foreign languages fluently.	0.85
	7. If Turkey had no contact with Chinese-speaking countries, it would be a great loss.	0.62
	21. I wish to get to know more native Chinese speakers.	0.74
	22. Learning Chinese is important because it enables me to meet and communicate with a diverse range of people.	0.53
	30. I am genuinely interested in learning multiple foreign languages.	0.76
3. Instrumentality	13. Learning Chinese is crucial for my career aspirations.	0.67
	14. Chinese is a global language.	0.61
	26. Learning Chinese will contribute to my overall education.	0.62
	39. Learning Chinese will enhance my job prospects.	0.76
	48. Learning Chinese is a growing trend worldwide.	0.60
4. Attitudes towards the learning situation	5. I early anticipated attending Chinese class because of my exceptional teacher.	0.65
	19. I really like my Chinese teacher.	0.60
	24. I enjoy the activities of my Chinese class very much.	0.64
	34. My Chinese teacher employs a dynamic and engaging instructional style.	0.73
	44. My Chinese teacher is a great source of inspiration for me.	0.74
5. Language anxiety	4. I often lack confidence when speaking in our Chinese class.	0.54
	12. I experience anxiety when someone asks me something in Chinese.	0.64
	18. I feel embarrassed to volunteer answers in our Chinese classes.	0.78
	25. Speaking Chinese in any setting makes me feel uneasy.	0.75
	38. It would bother me if I had to speak Chinese on the telephone.	0.80
	51. I feel uncomfortable speaking Chinese outside the classroom.	0.70
6. Parental encouragement	2. My parents try to help me to learn Chinese.	0.74
	16. My parents feel that it is very important for me to learn Chinese.	0.69
	31. My parents are very interested in everything I do in my Chinese classes.	0.81
	45. My parents believe that I should devote more time to learning Chinese.	0.68
7. Passive motivation	10. I learn Chinese because it is a required course.	0.67
	27. I learn Chinese because it is a compulsory course.	0.72
	41. I learn Chinese to meet my parents’ expectations.	0.52

**Table 4:** Comparison of L2 motivation across genders (*n* = 256).

	Mean	Standard Deviation	*t (df)*	*p*
Male (*n* = 108)	4.80	0.61	−4.45 (254)	0.000
Female (*n* = 148)	5.14	0.59		

**Table 5:** Independent Sample *t*-test for the seven motivational variables across genders (*n* = 256).

	Male (*n* = 108)		Female (*n* = 148)		*t*
	Mean	Standard Deviation	Mean	Standard Deviation	
ATLC	5.52	0.74	5.74	0.75	−2.403 *
INTE	5.96	0.83	6.22	0.61	−2.796 *
INST	5.43	0.93	5.64	0.92	−1.769
ATLS	5.85	0.98	5.97	0.86	−1.084
LA	3.53	1.11	3.91	1.33	−2.435 *
PE	4.28	1.42	4.97	1.44	−3.84 **
PM	3.06	1.65	3.54	1.83	−2.136 *

Note: ATLC = Attitudes towards learning Chinese, INTE = integrativeness, INST = instrumentality, ATLS = attitudes towards the learning situation, LA = language anxiety, PE = parental encouragement, PM = passive motivation, * *p* < 0.05; ** *p* < 0.01.

**Table 6:** Comparison of L2 motivation between Chinese majors and non-Chinese-major students (*n* = 256).

Overall L2 Motivation	Mean	Standard Deviation	*t (df)*	*p*
Chinese majors (*n* = 101)	5.21	0.62	−4.62 (254)	0.000
Non-Chinese major students (*n* = 155)	4.86	0.58		

**Table 7:** Comparison of motivational subscales across majors (*n* = 256).

	Chinese Majors (*n* = 101)		Non-Chinese Majors (*n* = 155)		*t*
	Mean	Standard Deviation	Mean	Standard Deviation	
ATLC	5.57	0.81	5.70	0.72	1.36
INTE	6.00	0.72	6.19	0.71	2.08 *
INST	5.52	0.99	5.57	0.88	0.47
ATLS	5.51	1.00	6.18	0.73	5.79 **
LA	4.15	1.30	3.49	1.16	−4.29 **
PE	5.31	1.12	4.27	1.53	−6.26 **
PM	4.44	1.59	2.62	1.49	−9.29 **

Note: ATLC = attitudes towards learning Chinese, INTE = integrativeness, INST = instrumentality, ATLS = attitudes towards the learning situation, LA = language anxiety, PE = parental encouragement, PM = passive motivation, * *p* < 0.05; ** *p* < 0.01.

## Data Availability

The data that support the findings of this study are available from the corresponding author upon reasonable request.
